# Testosterone and the Amygdala’s Functional Connectivity in Women and Men

**DOI:** 10.3390/jcm12206501

**Published:** 2023-10-13

**Authors:** Lydia Kogler, Veronika I. Müller, Ewald Moser, Christian Windischberger, Ruben C. Gur, Ute Habel, Simon B. Eickhoff, Birgit Derntl

**Affiliations:** 1 Department of Psychiatry and Psychotherapy, Tübingen Centre for Mental Health (TüCMH), Medical Faculty, University of Tübingen, Calwerstrasse 14, 72076 Tübingen, Germany; lydia.kogler@med.uni-tuebingen.de; 2 German Center for Mental Health (DZPG) Partner Site, 72076 Tübingen, Germany; 3 Institute of Neuroscience and Medicine: Brain and Behavior (INM-7), Research Centre Jülich, 52425 Jülich, Germany; v.mueller@fz-juelich.de (V.I.M.); s.eickhoff@fz-juelich.de (S.B.E.); 4 Institute of Systems Neuroscience, Medical Faculty, Heinrich Heine University Düsseldorf, Moorenstraße 5, 40225 Düsseldorf, Germany; 5 High-Field MR Center, Center for Medical Physics and Biomedical Engineering, Medical University of Vienna, Waehringer Guertel 18-20, 1090 Vienna, Austria; ewald.moser@meduniwien.ac.at (E.M.); christian.windischberger@meduniwien.ac.at (C.W.); 6 Brain Behavior Laboratory and Neurodevelopment and Psychosis Section, Department of Psychiatry, Perelman School of Medicine, University of Pennsylvania, Philadelphia, PA 19104, USA; gur@upenn.edu; 7 Department of Psychiatry, Psychotherapy and Psychosomatics, RWTH Aachen University, Pauwelsstrasse 30, 52074 Aachen, Germany; uhabel@ukaachen.de; 8 JARA BRAIN Institute I, Translational Brain Medicine, Forschungszentrum Jülich GmbH, 52425 Jülich, Germany; 9 LEAD Graduate School and Network, University of Tübingen, Walter-Simon-Straße 12, 72074 Tübingen, Germany; 10 International Max Planck Research School for the Mechanisms of Mental Function and Dysfunction (IMPRS-MMFD), Otfried-Müller-Str. 27, 72076 Tübingen, Germany

**Keywords:** testosterone, amygdala, frontal cortex, occipital cortex, sex, gender, personality, resting-state, functional connectivity, functional magnetic resonance imaging

## Abstract

The amygdala contains androgen receptors and is involved in various affective and social functions. An interaction between testosterone and the amygdala’s functioning is likely. We investigated the amygdala’s resting-state functional connectivity (rsFC) network in association with testosterone in 94 healthy young adult women and men (final data available for analysis from 42 women and 39 men). Across the whole sample, testosterone was positively associated with the rsFC between the right amygdala and the right middle occipital gyrus, and it further predicted lower agreeableness scores. Significant sex differences appeared for testosterone and the functional connectivity between the right amygdala and the right superior frontal gyrus (SFG), showing higher testosterone levels with lower connectivity in women. Sex further predicted the openness and agreeableness scores. Our results show that testosterone modulates the rsFC between brain areas involved in affective processing and executive functions. The data indicate that the cognitive control of the amygdala via the frontal cortex is dependent on the testosterone levels in a sex-specific manner. Testosterone seems to express sex-specific patterns (1) in networks processing affect and cognition, and (2) in the frontal down-regulation of the amygdala. The sex-specific coupling between the amygdala and the frontal cortex in interaction with the hormone levels may drive sex-specific differences in a variety of behavioral phenomena that are further associated with psychiatric illnesses that show sex-specific prevalence rates.

## 1. Introduction

The amygdalae are small almond-shaped bilateral anatomical structures that are involved in signaling the emotional salience of events [1,2]. They are major structures of the limbic system and drive various behavioral functions. Amygdala dysfunctions are associated with a variety of psychiatric illnesses that show sex-specific prevalence rates [3]. There is some evidence that BOLD-based amygdala activity reveals specific patterns in women and in men when modulating emotional and stress-related responses [4,5,6,7]. The human amygdala contains androgen receptors [8,9] that may be activated by testosterone, a steroid hormone involved in emotional and social behavior [10,11,12]. Testosterone is associated with social-oriented behavior such as extraversion [13,14]. The administration of testosterone has been shown to influence emotional and social functions, such as an increase in selfish behavior [15], a decrease in cognitive reflection in men [16], and changes in assertiveness [17].

The brain continuously exchanges information among structurally and functionally linked regions [18]. The temporally dependent neural activation patterns of anatomically separated regions are assumed to reflect functional communication throughout the brain. This effect—namely, functional connectivity [19,20]—is likely to play a key role during complex cognitive processes, but also during rest, as a large amount of spontaneous activity is highly correlated for multiple regions [21,22]. The amygdala is connected to multiple regions involved in the regulation of emotional and social information [23,24,25]. Due to this strong involvement in emotional and social behavior, the amygdala’s communication and integration of complex information are of high interest.

There is also evidence that functional connectivity is affected by sex [26,27,28,29,30] as well as by testosterone [31,32,33,34], although none of these studies investigated both the sex- and testosterone-specific effects of amygdala connectivity at rest. Nevertheless, reports on sex differences in amygdala activity [35,36], as well as in sex hormone levels [37], suggest that the functional connectivity of the amygdala is associated with the hormone levels in a sex-specific manner.

It was shown that testosterone modifies the amygdala’s functional connectivity, especially with the prefrontal cortex, when performing emotional tasks [38]. Peper and colleagues [38], in their review, conclude that androgens such as testosterone may decrease subcortico–cortical connectivity but increase connectivity between subcortical areas. It was further speculated that testosterone’s impact on behavior is modulated via the cognitive control of the frontal cortex over the amygdala [39]. Several studies show an association between testosterone and amygdala–prefrontal cortex coupling. In men, exogenous testosterone administration reduces the functional connectivity between the amygdala and the dorsolateral prefrontal cortex (DLPFC) [40]. In women, decreased connectivity between the amygdala and orbitofrontal regions after testosterone administration during emotional tasks is reported [39,41,42]. In addition, endogenous testosterone has shown effects on the amygdala’s connectivity. While the resting-state functional connectivity (rsFC) between the amygdala and orbitofrontal regions decreases with higher endogenous testosterone levels in male adolescents [43], in men, endogenous testosterone was significantly correlated with the task-based connectivity of the amygdala and the ventrolateral prefrontal cortex in an emotional approach–avoidance task [44]. Notably, in women, the endogenous testosterone level was inversely correlated with the coupling of the amygdala and the superior temporal gyrus [34], and changes in testosterone levels due to competition were associated with stronger coupling of the amygdala and the orbitofrontal cortex [34]. 

Summarizing these previous studies, endogenous testosterone seems to be associated with increased coupling of the amygdala with the frontal cortex in men but with decreased coupling between the amygdala and cortical regions in women. Therefore, an interaction between sex, testosterone, and functional connectivity is likely, and it can be hypothesized that testosterone reconfigures neural functional connectivity at rest in a sex-specific manner. This is specifically of interest as testosterone and amygdala functioning seem to drive social and affective behavior, albeit potentially differently in women and men. Sex differences are further evident in the prevalence rates of neurological and mental disorders (e.g., Parkinson’s disease, Huntington’s disease, dyslexia, attention deficit hyperactivity disorder, autism, depression, anxiety disorder, schizophrenia) [45,46,47,48]. However, until now, no direct comparison of the amygdala’s functional connectivity and its relationship to testosterone in women and men has been published. Unfortunately, research investigating associations with hormones often focuses either on female or male samples. Within this research domain, conclusions that are based on studying one sex only have limited value in understanding the same phenomena in the other sex [46]. Thus, the interaction of amygdala connectivity with hormone levels may contribute to sex differences in a variety of behavioral phenomena. 

We can only speculate about a sex-specific impact of testosterone on the amygdala’s functional connectivity. Combining previous reports, however, this is highly expected. RsFC allows for the revealing of the functional organization of the brain and contributes to the understanding of sex differences, the coupling between the amygdala and other brain regions, as well as its association with endogenous testosterone, independent of an applied task. 

Here, we examine the association of functional connectivity strength with testosterone levels. In particular, the aims of the current study are (1) to assess the rsFC of the amygdala in healthy young women and men in association with endogenous testosterone, and (2) to analyze whether women and men express different patterns of rsFC of the amygdala in association with testosterone. Based on previous findings, we hypothesize that higher endogenous testosterone levels are associated with increased connectivity of the amygdala with prefrontal regions in men [44], whereas testosterone is negatively associated with the connectivity of the amygdala and frontal regions in women [34,39,41]. Furthermore, as reports indicate an association between testosterone and social-oriented behavior, we expect that the functional connectivity of the amygdala in association with testosterone also impacts social-oriented personality traits [13,14,15,16,17].

## 2. Materials and Methods

### 2.1. Sample

In 94 students (non-smoking, right-handed), resting-state data and anatomical scans were assessed (see also [30] for a further description of the sample). Thirteen subjects were excluded (missing hormone data: *n* = 1; outliers in hormone data (mean +/− 2 standard deviations): *n* = 8; sickness: *n* = 1; scanner movement matching between groups: *n* = 3 [49,50]), resulting in a final sample of 81 participants (42 women; see Table 1 for the sample description and [30]). 

The exclusion criteria were any history of psychiatric or neurological disorders, chronic diseases (e.g., allergic asthma), drug intake, competitive sports, working night shifts, hormone intake, premenstrual dysphoric disorder, recent or current pregnancy, and any MR incompatibility. Only naturally cycling women without the use of oral contraception were included. The cycle length was estimated by tracking at least three previous cycles. Women with a cycle length of >35 days were excluded.

To increase comparability by decreasing the potential effects of the circadian rhythm, the measurements were scheduled in the afternoon between 2:30 p.m. and 5:30 p.m. Participants were asked to refrain from exercise or alcohol consumption (24 h prior to the measurements), medication, caffeine and drug intake (on the test day), and food and drink other than water (for two hours before the measurements). Upon arrival, participants were asked for subjective mood ratings (positive and negative affect scale, PANAS) [51] and to provide saliva samples for hormone analyses (approximately 15 min after arrival) (see also [30,52,53,54]). Furthermore, participants completed questionnaires on personality (NEO-FFI, [55]) and social gender roles (BSRI, [56]). 

This study was approved by the Institutional Review Board of the Medical University of Vienna (project number: 2011/1134). Participants were treated according to the Declaration of Helsinki (1964). Written informed consent was obtained from all participants.

### 2.2. Saliva Samples

The saliva samples were stored at −20 °C at least until shipping to the analysis laboratory (SwissHealthMed, Aying, Germany), where they were thawed and centrifuged. To obtain the testosterone concentrations, competitive luminescence immunoassay kits (LUMI) were used, which have minimal cross-reactivity with other steroid hormones and achieved reliable measurements (intra-assay CV < 4% and inter-assay CV < 7%). The lower sensitivity limit of the immunoassay kits was 1.8 pg/mL and the standard curve range was 1.8–500 pg/mL. Salivary testosterone measures correlate positively with serum-free testosterone levels and other circulating androgen markers (bioavailable testosterone, total testosterone) [57,58]. 

The hormone data were transformed using a log transformation (y = log10(x + 1)) prior to the statistical analyses as they were not normally distributed.

### 2.3. Data and Statistical Analysis of Behavioral and Hormone Data

For the statistical analyses of the sex differences in age, testosterone levels, PANAS, NEO-FFI, and BSRI, as well for the regression analyses, IBM SPSS Statistics for Windows, Version 20.0 (IBM Corp., Armonk, NY, USA) was used. The significance level was set to *p* < 0.05. The results were corrected for multiple comparisons (Bonferroni–Holm correction). To explore the associations of testosterone, functional connectivity and sex with social behavior, we performed exploratory multiple regression analyses with personality characteristics impacting social behavior as the dependent variable. Multiple linear regression analyses (predictors: sex, testosterone, and amygdala connectivity that appeared at the whole-brain level in association with testosterone, and their interactions; predictors were centered; forced entry) on personality (NEO-FFI) and social gender roles (BSRI) were performed to further characterize the impact of testosterone, sex and brain connectivity on social personality characteristics. As sex and testosterone were highly correlated, we ran separate analyses for these predictors. Thus, we ran multiple regression models with (a) sex and amygdala connectivity that appeared at the whole-brain level as predictors and the NEO-FFI and BSRI scales as the outcome variables; and (b) testosterone and amygdala connectivity that appeared at the the whole-brain level as predictors and the NEO-FFI and BSRI scales as the outcome variables.

### 2.4. Resting-State Functional Connectivity Analysis 

#### 2.4.1. Acquisition and Preprocessing

The resting-state data were acquired using T2*-weighted echo-planar imaging (EPI) with a 3 Tesla Tim Trio Scanner (Siemens Medical Systems, Erlangen, Germany) with the following parameters: 167 time points, 23 axial slices with interleaved acquisition, TE/TR = 38/1800 ms, voxel size 1.5 × 1.5 × 3 mm, bandwidth = 1446 Hz/pixel, 1.8 mm slice gap. The data were processed using SPM8 (Wellcome Trust Centre for Neuroimaging, London, http://www.fil.ion.ucl.ac.uk/spm/software/spm8/; accessed on 29 September 2023; MATLAB Version R2012b; MathWorks Inc., Sherborn, MA, USA). Prior to the analyses, the first four volumes were discarded. The images were corrected for geometric distortions using field maps. Head movement was corrected via affine registration using a two-pass procedure (images were first aligned to the initial volume and subsequently to the mean of all the volumes). Next, the mean EPI image was spatially normalized to the MNI152 template [59], i.e., via the “unified segmentation” approach [60]. The ensuing deformation was applied to the individual EPI volumes. The images were smoothed (5 mm full-width-at-half-maximum Gaussian kernel) to improve the signal-to-noise ratio and to compensate for any residual anatomical variations. The data were processed as follows [30,61]. In order to reduce the spurious correlations, the voxel time-series were regressed against the following nuisance variables: (1) six motion parameters (image realignment), and (2) their first derivatives. All the nuisance variables entered the model as first- and second-order terms [50]. The data were further band-pass filtered (cut-off frequencies of 0.01 and 0.08 Hz). 

#### 2.4.2. Functional Connectivity Analyses

The amygdala volumes (whole left and right volumes), as seed regions, were derived from the AnatomyToolbox v2.0 [62]. The time courses of all the voxels within each seed were extracted for each subject and expressed as the first eigenvariate. To quantify the rsFC, linear (Pearson) correlation coefficients were computed between the seed regions’ time series and the time series of all the other GM voxels of the brain. The voxel-wise correlation coefficients of each subject and seed were transformed into Fisher’s Z-scores. These were subjected to a second-level GLM to test for group differences and the effects of testosterone, with the factor sex and the covariate testosterone, including non-sphericity correction as implemented in SPM. 

Whole-group analyses (women and men; *n* = 81) of the testosterone associations were conducted in conjunction with the main effect of the amygdala’s (positive and negative) functional connectivity to restrict the analyses to only those regions that are significantly connected with the amygdala. Sex differences (men vs. women and women vs. men) in the pattern of the covariate testosterone were assessed in conjunction with the main effect of the amygdala (positive or negative rsFC) and masked with the main effect of the covariate testosterone for each sex. We determined the conjunctions between (1) sex differences in the pattern of the covariate testosterone with the association of the rsFC of the amygdala (t-tests between women and men) and (2) the main effect of the rsFC of the amygdala for each sex (testosterone x sex 1 vs. sex 2 ∩ amygdala). This conjunction-based approach tests for (a) sex differences in the pattern of the association between testosterone and the rsFC of the amygdala and (b) restricts the analyses in association with testosterone to regions that have significant functional connectivity with the amygdala. In addition to this, the conjunction was masked with the effects of testosterone in each sex (correlations for sex 1 or sex 2). This approach (a) restricts the analyses to regions that are significantly functionally connected with the amygdala and (b) shows whether the rsFC is associated with testosterone within women or men [30].

The results were thresholded at the cluster level of FWE *p* < 0.05 (cluster-forming threshold at the voxel level *p* < 0.001) [30]. For illustration purposes and to assess the direction of the sex differences in the correlations of testosterone and the rsFC, the correlation coefficient of the rsFC of the amygdala with regions that appeared to be significant at a whole-brain level were extracted and further investigated. 

### 2.5. Functional Characterization

To characterize the functions of the specific brain regions derived in the current analyses, we used the BrainMap database, which includes metadata of published functional neuroimaging experiments with coordinate-based results [63]. We used the metadata of functional neuroimaging experiments in BrainMap to identify the behavioral phenomena underlying the occurring regions. Importantly, this characterization reveals the behavioral domains and paradigm classes in which the regions of interests are typically involved. Thus, instead of basing our interpretation and discussion of the resulting brain regions on a few previous, manually selected studies that report on this region, we systematically tested across the published literature the tasks and paradigms that have been used when the respective region has been reported to be activated. Metadata categories in the BrainMap database that classify every single experimental contrast according to the “behavioral domain” (e.g., emotion, cognition, or perception) and “paradigm class” (e.g., flanker task, mental rotation tasks, or reward tasks) [64] were included (see http://brainmap.org/taxonomy/overview.html, accessed on 29 September 2023, for the complete list of behavioral domains and paradigm classes). The forward- and reverse-inference approaches were calculated for the analyses, as described previously [65,66]. The forward-inference approach determines the probability of observing activity in a brain region when a mental process is present. Thus, we tested whether the conditional probability of activation given a particular task P(Activation|Task) was higher than the baseline probability of activation P(Activation). The baseline denotes the probability of finding an (random) activation from BrainMap in the region of interest. Significance was tested using a binomial test (*p* < 0.05, corrected for the false discovery rate (FDR)). Additionally, the reverse-inference approach tests the probability of the presence of a mental process given knowledge of activation in a particular region of interest. This likelihood P(Task|Activation) can be derived from P(Activation|Task) as well as P(Task) and P(Activation) using Bayes’ rule. Significance was assessed by means of a chi-square test (*p* < 0.05, corrected for multiple comparisons).

### 2.6. Voxel-Based Morphometry (VBM) Analysis

To exclude the potential effects between testosterone and the amygdala volume, we performed a structural covariance analysis. A high-resolution anatomical image with a T1w-MPRAGE sequence (3-D Magnetization Prepared Rapid Gradient Echo: 160 sagittal slices, TR = 2300 ms, TE = 4.21 ms, 1 × 1 × 1.1 mm resolution, flip angle 9°, inversion time 900 ms) was acquired from each participant. The anatomical scans were preprocessed with the VBM8 toolbox (dbm.neuro.uni-jena.de/vbm) in SPM8 using standard settings (DARTEL normalization, spatially adaptive non-linear means denoising). Within a unified segmentation model [60], the images were corrected for bias-field inhomogeneities. The brain tissue was classified into gray matter, white matter and cerebrospinal fluid, adjusted for any partial volume effects and spatially normalized to the Montreal Neurological Institute (MNI) template. The segmented images were non-linearly modulated to adjust them to the amount of expansion and contraction, which was applied during normalization. We computed the volume of the left and right amygdala by integrating the (non-linearly) modulated voxel-wise gray matter probabilities for each subject. Age was included as a nuisance variable. As we did not multiply the segmented images by the linear components but rather modulated the images by the non-linear components only, the calculated gray matter volume represents the amount of gray matter corrected for the individual brain size (see also [30] for the VBM analysis). We performed a structural covariance analysis to test for potential effects between testosterone and the amygdala volume. Furthermore, we tested for sex differences in the correlations of testosterone with the left or right amygdala volume. Statistical significance was evaluated at *p* < 0.05 and Bonferroni-corrected for multiple comparisons.

## 3. Results

### 3.1. Sample Description

**Figure 1:**
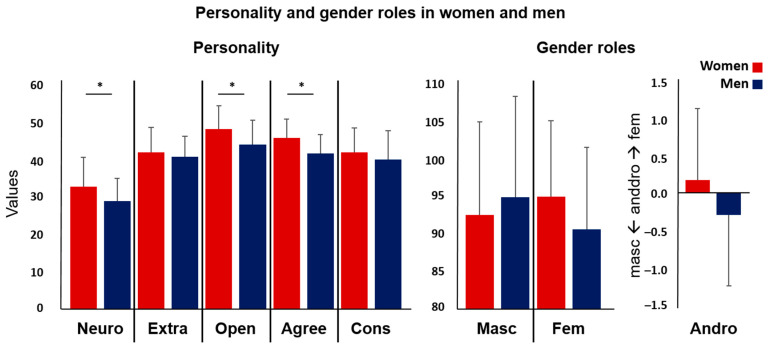
Personality (NEO-FFI) and gender role (BSRI) values separately for women and men. Significant sex differences appeared for neuroticism, openness, and agreeableness (marked with an *). (Neuro = neuroticism, Extra = extraversion, Open = openness, Agree = agreeableness, Cons = conscientiousness, Masc = masculinity, Fem = femininity, Andro = androgyny).

The women and men did not differ in age, positive or negative affect (PANAS) (see Table 1), nor in the resting-state movement parameters (DVARS, FD, RMD, all *p*s > 0.689). The men had significantly higher testosterone levels than the women (t(79) = 8.401, *p* < 0.001, d = 1.868), whereas the women had higher scores for neuroticism (t(79) = 2.456, *p* = 0.016, d = 0.546), openness (t(79) = 2.936, *p* = 0.004, d = 0.653), and agreeableness (t(79) = 3.760, *p* < 0.001, d = 0.836) (Figure 1).

### 3.2. Amygdala rsFC and Testosterone: Whole Group

**Figure 2:**
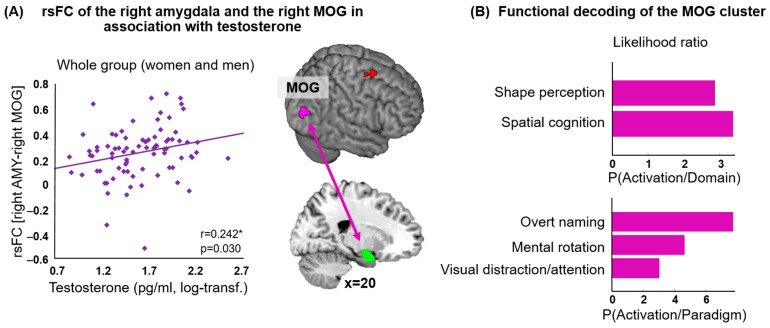
(**A**) Higher testosterone levels were associated with stronger resting-state functional connectivity (rsFC) between the right amygdala (green) and the right middle occipital gyrus (MOG, violet) over the whole group (women and men) (The significant association is marked with an *, log-transf. = log-transformed). (**B**) Likelihood ratio for the forward-inference approach for significant behavioral domains (graphs in the upper panel) and paradigm classes (graphs in the lower panel) for the cluster in the MOG showing a significant rsFC with the amygdala in association with testosterone.

Across the whole group, higher testosterone levels were associated with stronger functional connectivity between the right amygdala and the right middle occipital gyrus (MOG) (as shown in Figure 2A, Table 2). The functional decoding of the right MOG linked this region with visual perception of shape and spatial cognition (behavioral domain, forward inference) and overt naming, mental rotation and visual distraction and attention (paradigm classes, forward inference) (Figure 2B). For the reverse-inference approach, attention emerged additionally.

### 3.3. Amygdala rsFC and Testosterone: Men vs. Women

**Figure 3:**
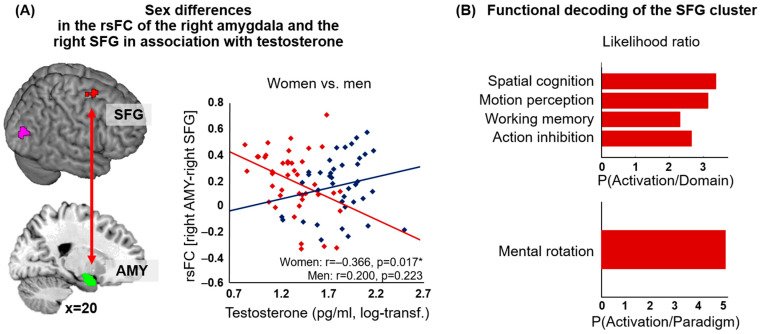
(**A**) We found a significant negative correlation of testosterone with the resting-state functional connectivity (rsFC) between the right amygdala (AMY, green) and the right superior frontal gyrus (SFG, red) in women (red dots, red line) (marked with an *). Higher testosterone levels were associated with decreased rsFC of the amygdala and the SFG. In men (blue dots, blue line), no significant association was seen (log-transf. = log-transformed). (**B**) Likelihood ratio for the forward-inference approach for the significant behavioral domains (graph in the upper panel) and paradigm classes (graph in the lower panel) for the cluster in the SFG showing sex differences in the association between testosterone and the rsFC of the amygdala.

The men and women differed in the relationship of testosterone and the functional connectivity of the right amygdala with the right posterior superior frontal gyrus (SFG) (as shown in Figure 3A, Table 2). In women, lower testosterone levels were associated with increased rsFC between the amygdala and the right SFG. No significant association, albeit the reverse pattern (higher testosterone levels going along with increased rsFC), was seen for men. No significant effects were seen for the left amygdala. Functional decoding revealed an association between the cluster in the right posterior SFG and the behavioral domains of spatial cognition, motion perception, working memory and action inhibition as well as the paradigm class of mental rotation (forward inference) (Figure 3B). For the reverse-inference approach, additionally, the behavioral domain of action execution and the paradigm classes of *n*-back and saccades appeared.

### 3.4. Exploratory Regression Analyses: Impact on Social Behavior

Multiple regression analyses with the predictors sex, rsFC (right amygdala—right MOG) and rsFC (right amygdala—right SFG) revealed the following results. Significant effects appeared for sex on openness (model R^2^ = 0.150, *p* = 0.029, β = 0.305, *p* = 0.006) and on agreeableness (model R^2^ = 0.171, *p* = 0.014, β = 0.381, *p* = 0.001). No other effects appeared for the other NEO-FFI scales (all *p*s > 0.111). For social gender roles, no significant models appeared (all *p*s > 0.243). 

**Figure 4:**
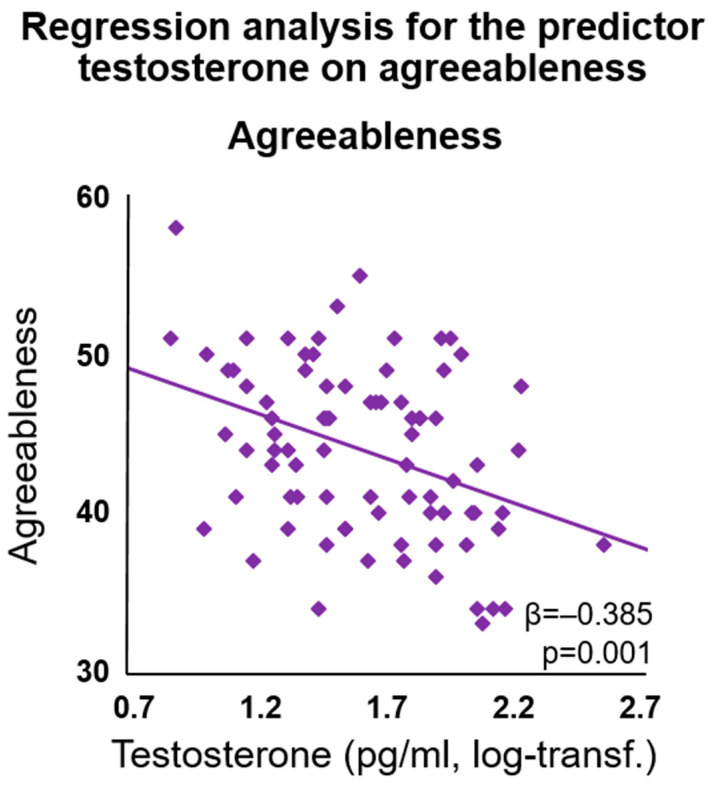
Testosterone was a significant predictor of agreeableness in the exploratory regression analysis.

Multiple regression analyses with the predictors testosterone, rsFC (right amygdala—right MOG) and rsFC (right amygdala—right SFG) revealed the following results. Testosterone had a significant effect on agreeableness (model R^2^ = 0.169, *p* = 0.015; β = −0.385, *p* = 0.001), with higher testosterone levels indicating lower scores for agreeableness (see Figure 4). No other effects on the NEO-FFI scales appeared (all *p*s > 0.244). No significant models for social gender roles appeared (all *p*s > 0.482). 

Please see Tables S1 and S2 in the Supplementary Material for further details on the exploratory regression analyses.

### 3.5. VBM Analysis

No significant correlation occurred between the gray matter volume of the left or right amygdala and the testosterone levels in women and men (all *p*s > 0.134). 

## 4. Discussion

The aim of the current study was to examine whether sex differences in the amygdala’s functional whole-brain connectivity are associated with testosterone levels and whether the rsFC strength is further associated with social personality traits. Sex differences are evident in various cognitive and emotional domains, as well as in mental health issues, including the diverging prevalence rates of neurological and mental disorders in women and men (e.g., Parkinson’s disease, Huntington’s disease, dyslexia, attention deficit hyperactivity disorder, autism, depression, anxiety disorder, schizophrenia) [45,46,47,48]. Likewise, sex differences appear in testosterone expression [53,67,68,69], as well as in the expression of the androgen receptor [70], which may contribute to mental or neurological disorders with sex-specific prevalence rates [71,72]. Assessing the sex-specific association between testosterone and the amygdala’s network is therefore of high interest. 

### 4.1. Testosterone and Functional Connectivity of the Right Amygdala

In accordance with previous findings [44], our results show that the testosterone levels are associated with the amygdala’s functional connectivity. Across women and men, we observed a positive association between testosterone and the functional connectivity of the right amygdala with the right middle occipital gyrus (MOG). The right hemisphere is dominant for visuospatial attention and memory (for a review on lateralization, see, e.g., [73]), testosterone seems to mediate lateralization [74,75], and the lateralization of the right occipital lobe during visual attention is further associated with testosterone [74]. Notably, in contrast to our previous study assessing the association of the amygdala’s connectivity with cortisol [30], the amygdala’s connectivity in association with testosterone is right-lateralized, whereas for cortisol it mainly seems left-lateralized. 

Assessing the functional connectivity in the “resting brain” has the advantage of exploring group differences independent of an employed task, which might be affected by group differences in performance. We therefore relied on functional decoding analyses via indirect inferences for the interpretation of specific functions of the occurring clusters showing the rsFC with the amygdala (MOG, SFG). These functional decoding analyses of the brain areas provide the opportunity to further characterize the respective regions in a quantitative manner. Thus, our use of the BrainMap database augments the interpretation of the neuroimaging outcomes. Based on this functional characterization analysis and the forward-inference approach, the observed part of the MOG in the current study is involved in the paradigm class of visual attention and the behavioral domain of spatial cognition, amongst others. Indeed, it was shown that exogenous testosterone enhances attention in men [76], and also performance in mental rotation tasks in women [77] and men [78], as well as the amygdala’s activation during spatial navigation [77]. Increased testosterone is further associated with visual attention [79,80]. These results align with the outcome of our study. Our data further revealed lower agreeableness scores with higher testosterone levels. Associations between personality traits and testosterone are reported frequently [13,14,81]. Metzger and Boettger, for instance, reported changes in assertiveness after testosterone therapy in transgender men [17]. It was also shown that testosterone administration increases selfish behavior in economic decisions [15]. Taken together, these results indicate that higher testosterone levels go along with less external social orientation but higher self-focus. Focused behavior and attention induced by testosterone might trigger increased emotional awareness in relation to spatial cognition, which is reflected in the enhanced coupling of the amygdala and the MOG in our study. It seems that, in general, testosterone increases the linkage between emotional salience and spatial processing. In addition, the association between mental rotation and the testosterone levels disappears after mental rotation training in women and men [82], suggesting that testosterone rather increases the emotional salience of cognitive processing than improving performance per se.

### 4.2. Sex Differences

We observed a negative association between testosterone and the functional connectivity between the right amygdala and right SFG (dorsolateral prefrontal cortex, DLPFC) exclusively in women. The SFG cluster overlaps with the premotor cortex [83]. A positive association between its activation and the testosterone levels during pain stimulation in men [84], and its functional connectivity to the amygdala [85,86], were previously reported. Our results are also in line with previous studies reporting the decreased coupling of the amygdala with cortical regions in women with higher testosterone [34,39,41]. In men, the findings are controversial, with a stronger coupling between the amygdala with cortical regions and endogenous testosterone [44] but the opposite effect with exogenous testosterone [40]. Additionally, we previously reported a similar sex-specific pattern for the association between cortisol and amygdala–SFG coupling [30]. It was speculated that testosterone’s impact on social and emotional behavior is modulated via the cognitive control of the frontal cortex over the amygdala [39]. Indeed, some evidence shows that the functional connectivity between the frontal cortex and the amygdala is associated with testosterone (e.g., [40,44,87]). Our data indicate that this association differs between women and men for the SFG.

The forward-inference approach of the functional decoding analyses revealed the involvement of the SFG cluster in executive functions such as the behavioral domains of working memory and action inhibition, which was reported previously [83]. Thus, in men, testosterone might induce higher cognitive control of the amygdala via the frontal cortex, whereas in women, testosterone might reduce the cognitive inhibition of the amygdala’s functions. Experimental studies of neuromodulation support this notion. Transcranial direct current stimulation (tDCS) applied to the frontal cortex can decrease the amygdala’s activity [88] and, thereby, may additionally increase a flow experience in men [88]. Thus, decreased amygdala activation modulated by the frontal cortex may lead to more focused and controlled behavior in men. It needs to be further elucidated whether and how tDCS impacts testosterone levels or is modulated by hormonal levels, whether this effect is also seen for the superior lateral regions, and whether similar effects can be observed in women. There is systematic evidence that sex and sex hormones impact the effects of non-invasive brain stimulation such as tDCS [89,90]. Although testosterone is known to impact the activity and connectivity of brain regions (e.g., [91,92]), it needs to be elucidated in more detail which effects it has on treatments such as brain stimulation. Although an impact of lateral frontal cortex activation on amygdala activation is assumed, individual testosterone fingerprints might be significant, contributing biomarkers that impact the outcomes of, e.g., brain stimulation. Notably, although we relied on analyzing sex differences in the functional connectivity of the resting brain, independent of an applied task, the forward-inference approach of the behavioral decoding analysis showed an association of the occurring SFG cluster with a sex-sensitive paradigm class—namely, mental rotation—and the behavioral domain of spatial cognition. There is evidence that women and men differ in the strategies employed to solve mental rotation tasks [93]. Women tend to use analytic strategies, whereas men rely on holistic strategies [94], which is also reflected in diverging neural activation patterns [95]. Additionally, sex hormones seem to be differently associated with task performance in women and men. It seems that, in men, testosterone levels predict better task performance in a mental rotation task, which was not observed in women. In women, evoking gender stereotypes predicted a lower task performance, although this was not associated with testosterone levels [96]. Moreover, there is some evidence that testosterone affects mental rotation performance in women and men differently. While men exhibit the best performance at medium levels of testosterone, women perform best at high levels [71,82]. Taken together with our results, and considering the psycho–bio–social approach [96], the connectivity between the frontal cortex and amygdala might drive opposite effects in women and men, and it might decrease cognitive, frontal control of the amygdala in women while increasing it in men. Hence, testosterone might modulate the cognitive control mechanisms of the frontal cortex over the amygdala differently in women and men. Whether the levels of testosterone and neural functional connectivity impact these behaviors in women and men in a sex-specific manner remains to be tested.

### 4.3. Future Directions and Limitations

While we aimed to assess sex differences, we should emphasize that the similarities across women and men are high and the differences between the sexes are more subtle [97]. The exploration of the small effect sizes of the mean differences is nevertheless necessary to improve knowledge of sex-specific processes, patterns, and sex-sensitive disorders, including diagnoses and treatments, and to further enable sex-sensitive neuroscience and precision medicine in the future. The current results suggest future clinical opportunities to optimize and personalize medicine and to provide and choose the best intervention available for each individual. Furthermore, the associations between individual testosterone profiles, the brain’s functional organization, and treatments such as brain stimulation, psychopharmacological treatments, or psychotherapeutic interventions need to be investigated in more detail in the future to further improve and advance targeted therapies. 

This study has several limitations. The sample size is relatively small to run sex comparisons. Future studies should increase the sample sizes with available hormone data to guarantee robust and generalizable results. Naturally cycling women throughout the menstrual cycle were investigated (22 women were tested in the luteal phase, 15 during the follicular phase and 5 during ovulation). However, we did not analyze the data according to the phase of the menstrual cycle. For exploratory reasons, we compared the three groups regarding the testosterone levels and did not find any group differences (all *p*s > 0.174). However, we cannot exclude that the diverging progesterone and estrogen levels may interact with the reported findings regarding the testosterone effects (cf., [80]). Within the central nervous system, testosterone is aromatized to estrogens [98], and it cannot be excluded that increased levels of estradiol further exert effects on the brain’s functional architecture. The current study cannot assess this association as we do not have data on estradiol in men. Furthermore, the enzyme 5alpha-dehydrogenase, which converts testosterone into the more active form dihydro-testosterone, shows a higher affinity for progesterone than for testosterone [99]. Thus, high levels of progesterone during the luteal phase would, e.g., lead to less active testosterone [80,93]. The complex interaction of testosterone and progesterone remains to be elucidated, as it potentially could explain the divergent effects of testosterone on performance in women and men [71,82]. Future studies should also investigate the interaction of sex hormones within the central nervous system across the hormonal transition phases throughout the lifespan and the potential impact they have on the observed patterns. In this vein, also endocrine disorders such as polycystic ovary syndrome (PCOS) and its association with functional connectivity and testosterone should be considered. One of the characterizations of PCOS, which is the most common endocrine disorder in women of reproductive age, is hyperandrogenism [100]. To investigate the association between the functional connectivity of the amygdala and testosterone levels in women diagnosed with PCOS would be of high interest to further elucidate the effects that testosterone exerts on the brain’s functional organization. 

The current results revealed the influence of testosterone on the functional organization of the brain in women and men, and thus, they contribute to the understanding of sex differences independent of specific task demands. The rsFC has the advantage of assessing sex differences, the coupling between the amygdala and other brain regions, as well as its association with endogenous testosterone, independent of an applied task. In contrast, functional task-based data might induce sex-specific differences, which further can be associated with confounding variables. The amygdala is strongly involved in social behavior [4,5,6,7] and in networks associated with the regulation of affective and social information [23,24,25], and it contains androgen receptors [8,9] that might be activated by testosterone. The coupling of the amygdala with the frontal regions has been reported to differ between men and women (e.g., [40,41,42]). Testosterone is known to impact behavior and to vary with task demands, which is associated with sex differences [52,53]. There is evidence that testosterone impacts and regulates the function and organization of the brain (e.g., [91,92]). Thus, it might be that testosterone reconfigures functional networks in a sex-specific manner. Sex differences have been reported in the performance of various tasks, such as some tapping verbal, or visuospatial abilities (e.g., [96]). These sex differences might further be affected by confounding variables such as gender stereotypes [96,101] or time limits [102]. The functional organization of the brain seems to be dynamic, associated with endogenous and exogenous sex hormones (e.g., [103,104,105]), and related to further confounding variables such as stress, social behavior, environment, or genotype [106,107]. Testosterone seems to affect variables such as risk-taking behavior, aggression, and sexuality (e.g., [10,108]), which might further be associated with confounding variables such as socio-economic background. This complex interplay needs to be systematically elucidated to establish individual fingerprints and to further improve and advance individualized treatments for disorders affecting the brain’s architecture. To understand the underlying mechanisms, preclinical studies are relevant. In animals, task demands can modify the expression of androgen receptors in the amygdala, which further seems to be associated with social behavior [106]. We can only speculate on the sex-sensitive expression of androgen receptors due to environmental demands, although it might be that social and cognitive challenges differently impact the expression of androgen receptors in the central nervous system. In general, more integrative research is needed in the future, combining task-based and resting-state functional data with the assessment of additional variables that might impact the constructs under investigation, to further enhance the reliability and validity of the current results. Additionally, we want to acknowledge that additional confounding variables such as age or socio-economic status might impact the sex-sensitive dynamics that we observed between testosterone and functional connectivity.

## 5. Conclusions

The resting-state functional connectivity of the amygdala with the MOG and SFG is associated with the testosterone levels and partly differs between women and men. The levels of testosterone are positively associated with amygdala–MOG coupling and further predict the agreeableness scores across women and men. Sex predicts the openness and agreeableness scores, and the levels of testosterone are negatively correlated with the functional connectivity of the amygdala and the SFG exclusively in women, whereas this association trends toward positive, although not significant, in men. This SFG cluster is involved with working memory, action inhibition, and spatial cognition. The data might indicate that the cognitive control of the amygdala via the frontal cortex depends on the testosterone levels in a sex-specific manner. The coupling between the amygdala and the frontal cortex in interaction with the hormone levels may drive sex-specific differences in a variety of behavioral phenomena.

## Figures and Tables

**Table 1:** Sample description.

	Women		Men		
	Mean	STD	Mean	STD	*p*-Value
Age	24.31	3.942	24.00	3.052	0.695
Testosterone (pg/mL, log-transformed)	1.37	0.257	1.86	0.267	<0.001 *
Testosterone (pg/mL)	26.63	17.26	84.45	56.45	<0.001 *
Affect (PANAS, mean scores)					
Positive affect	2.63	0.708	2.64	0.625	0.952
Negative affect	1.25	0.355	1.19	0.222	0.362
Personality (NEO-FFI)					
Neuroticism	32.83	7.880	28.95	6.177	0.016 *
Extraversion	42.10	6.585	40.82	5.562	0.351
Openness	48.33	6.234	44.15	6.576	0.004 *
Agreeableness	45.98	5.033	41.77	5.029	0.000 *
Conscientiousness	42.07	6.535	40.08	7.727	0.212
Gender roles (BSRI)					
Masculinity	92.67	12.491	95.05	13.504	0.412
Femininity	95.14	10.166	90.79	10.931	0.067
Androgyny	0.18	0.979	−0.30	0.974	0.030 +

Note: Negative affect and testosterone raw scores were not normally distributed and were therefore log-transformed. For negative affect and raw scores of testosterone, Mann–Whitney U tests for independent samples were used to compare groups. Significant group differences are marked with an *. + indicates results do not survive correction for multiple comparisons. STD = standard deviation.

**Table 2:** Connectivity of the right amygdala.

	*t* Value	X	Y	Z	Macroanatomical Location
(A) rsFC of the right amygdala in correlation with testosterone					
Whole group (*n* = 81)					
Cluster 1 (k = 98)					
	4.29	38	−82	14	R Middle occipital gyrus
	4.16	34	−74	10	
	3.91	42	−86	10	R Middle occipital gyrus
(B) rsFC of the right amygdala in correlation with testosterone					
Men (*n* = 39) > women (*n* = 42)					
Cluster 1 (k = 86)					
	4.90	24	6	62	R Superior frontal gyrus
	4.29	20	8	58	
	3.83	28	−2	62	

Note. Resting-state functional connectivity (rsFC) of the right amygdala. (A) rsFC of the right amygdala in association with testosterone (whole group, women and men). (B) Sex differences in the correlation between testosterone and the rsFC of the right amygdala. k = cluster size, R = right.

## Data Availability

Due to missing consent forms for public data-sharing, the data presented in this study are available on request from the corresponding author.

## Supplementary Materials

The following supporting information can be downloaded at https://www.mdpi.com/article/10.3390/jcm12206501/s1, Table S1. Multiple linear regressions (standardized beta coefficients) for sex, resting-state functional connectivity (rsFC) of the right amygdala with the right MOG and the right SFG, and their interactions (IA), as predictors of social behavior. Table S2. Multiple linear regressions (standardized beta coefficients) for testosterone (testo), resting-state functional connectivity (rsFC) of the right amygdala with the right MOG and the right SFG, and their interactions (IA), as predictors of social behavior (NEO-FFI, BSRI).

## References

[1] LeDoux J.E. (2000). Emotion Circuits in the Brain. Annu. Rev. Neurosci..

[2] Veer I.M., Oei N.Y.L., Spinhoven P., van Buchem M.A., Elzinga B.M., Rombouts S.A.R.B. (2011). Beyond Acute Social Stress: Increased Functional Connectivity between Amygdala and Cortical Midline Structures. Neuroimage.

[3] Gilpin N.W., Herman M.A., Roberto M. (2015). The Central Amygdala as an Integrative Hub for Anxiety and Alcohol Use Disorders. Biol. Psychiatry.

[4] Stevens J.S., Hamann S. (2012). Sex Differences in Brain Activation to Emotional Stimuli: A Meta-Analysis of Neuroimaging Studies. Neuropsychologia.

[5] Domes G., Schulze L., Böttger M., Grossmann A., Hauenstein K., Wirtz P.H., Heinrichs M., Herpertz S.C. (2010). The Neural Correlates of Sex Differences in Emotional Reactivity and Emotion Regulation. Hum. Brain Mapp..

[6] Kogler L., Gur R.C., Derntl B. (2015). Sex Differences in Cognitive Regulation of Psychosocial Achievement Stress: Brain and Behavior. Hum. Brain Mapp..

[7] McRae K., Ochsner K.N., Mauss I.B., Gabrieli J.J.D., Gross J.J. (2008). Gender Differences in Emotion Regulation: An FMRI Study of Cognitive Reappraisal. Group Process. Intergroup Relat..

[8] Simerly R.B., Chang C., Muramatsu M., Swanson L.W. (1990). Distribution of Androgen and Estrogen Receptor MRNA-Containing Cells in the Rat Brain: An In Situ Hybridization Study. J. Comp. Neurol..

[9] De Kloet E.R., Vreugdenhil E., Oitzl M.S., Joëls M. (1998). Brain Corticosteroid Receptor Balance in Health and Disease. Endocr. Rev..

[10] Eisenegger C., Haushofer J., Fehr E. (2011). The Role of Testosterone in Social Interaction. Trends Cogn. Sci..

[11] Dickerson S.S., Kemeny M.E. (2004). Acute Stressors and Cortisol Responses: A Theoretical Integration and Synthesis of Laboratory Research. Psychol. Bull..

[12] Derntl B., Windischberger C., Robinson S., Kryspin-Exner I., Gur R.C., Moser E., Habel U. (2009). Amygdala Activity to Fear and Anger in Healthy Young Males Is Associated with Testosterone. Psychoneuroendocrinology.

[13] Afrisham R., Sadegh-Nejadi S., SoliemaniFar O., Kooti W., Ashtary-Larky D., Alamiri F., Aberomand M., Najjar-Asl S., Khaneh-Keshi A. (2016). Salivary Testosterone Levels under Psychological Stress and Its Relationship with Rumination and Five Personality Traits in Medical Students. Psychiatry Investig..

[14] Smeets-Janssen M.M.J., Roelofs K., Van Pelt J., Spinhoven P., Zitman F.G., Penninx B.W.J.H., Giltay E.J. (2015). Salivary Testosterone Is Consistently and Positively Associated with Extraversion: Results from the Netherlands Study of Depression and Anxiety. Neuropsychobiology.

[15] Wu Y., Liao J., Zilioli S., Wu Y., Deng H., Li H., Tobler P.N. (2019). Testosterone Administration Increases Social Discounting in Healthy Males. Psychoneuroendocrinology.

[16] Nave G., Nadler A., Zava D., Camerer C. (2017). Single-Dose Testosterone Administration Impairs Cognitive Reflection in Men. Psychol. Sci..

[17] Metzger N.Y., Boettger S. (2019). The Effect of Testosterone Therapy on Personality Traits of Trans Men: A Controlled Prospective Study in Germany and Switzerland. Psychiatry Res..

[18] Eickhoff S.B., Müller V.I. (2015). Functional Connectivity. Brain Mapping—An Encyclopedic Reference. Volume 2: Anatomy and Physiology, Systems.

[19] Eickhoff S.B., Grefkes C. (2011). Approaches for the Integrated Analysis of Structure, Function and Connectivity of the Human Brain. Clin. EEG Neurosci..

[20] Friston K.J. (2011). Functional and Effective Connectivity: A Review. Brain Connect..

[21] Buckner R.L., Vincent J.L. (2007). Unrest at Rest: Default Activity and Spontaneous Network Correlations. Neuroimage.

[22] Greicius M.D., Krasnow B., Reiss A.L., Menon V. (2003). Functional Connectivity in the Resting Brain: A Network Analysis of the Default Mode Hypothesis. Proc. Natl. Acad. Sci. USA.

[23] Robinson J.L., Laird A.R., Glahn D.C., Lovallo W.R., Fox P.T. (2011). Meta-Analytic Connectivity Modeling: Delineating the Functional of the Human Amygdala. Hum. Brain Mapp..

[24] Roy A.K., Shehzad Z., Margulies D.S., Kelly A.M.C., Uddin L.Q., Gotimer K., Biswal B.B., Castellanos F.X., Milham M.P. (2010). Functional Connectivity of the Human Amygdala Using Resting State FMRI. Neuroimage.

[25] Stein J.L., Wiedholz L.M., Bassett D.S., Weinberger D.R., Zink C.F., Mattay V.S., Meyer-Lindenberg A. (2007). A Validated Network of Effective Amygdala Connectivity. Neuroimage.

[26] Biswal B.B., Mennes M., Zuo X.-N., Gohel S., Kelly C., Smith S.M., Beckmann C.F., Adelstein J.S., Buckner R.L., Colcombe S. (2010). Toward Discovery Science of Human Brain Function. Proc. Natl. Acad. Sci. USA.

[27] Satterthwaite T.D., Wolf D.H., Roalf D.R., Ruparel K., Erus G., Vandekar S., Gennatas E.D., Elliott M.A., Smith A., Hakonarson H. (2014). Linked Sex Differences in Cognition and Functional Connectivity in Youth. Cereb. Cortex.

[28] Wu K., Taki Y., Sato K., Hashizume H., Sassa Y., Takeuchi H., Thyreau B., He Y., Evans A.C., Li X. (2013). Topological Organization of Functional Brain Networks in Healthy Children: Differences in Relation to Age, Sex, and Intelligence. PLoS ONE.

[29] Wang L., Shen H., Tang F., Zang Y., Hu D. (2012). Combined Structural and Resting-State Functional MRI Analysis of Sexual Dimorphism in the Young Adult Human Brain: An MVPA Approach. Neuroimage.

[30] Kogler L., Müller V.I., Seidel E.-M., Boubela R., Kalcher K., Moser E., Habel U., Gur R.C., Eickhoff S.B., Derntl B. (2016). Sex Differences in the Functional Connectivity of the Amygdalae in Association with Cortisol. Neuroimage.

[31] Fareri D.S., Gabard-Durnam L., Goff B., Flannery J., Gee D.G., Lumian D.S., Caldera C., Tottenham N. (2015). Normative Development of Ventral Striatal Resting State Connectivity in Humans. Neuroimage.

[32] Vaisvaser S., Lin T., Admon R., Podlipsky I., Greenman Y., Stern N., Fruchter E., Wald I., Pine D.S., Tarrasch R. (2013). Neural Traces of Stress: Cortisol Related Sustained Enhancement of Amygdala-Hippocampal Functional Connectivity. Front. Hum. Neurosci..

[33] Sripada R.K., Swain J.E., Evans G.W., Welsh R.C., Liberzon I. (2014). Childhood Poverty and Stress Reactivity Are Associated with Aberrant Functional Connectivity in Default Mode Network. Neuropsychopharmacology.

[34] Buades-Rotger M., Engelke C., Krämer U.M. (2019). Trait and State Patterns of Basolateral Amygdala Connectivity at Rest Are Related to Endogenous Testosterone and Aggression in Healthy Young Women. Brain Imaging Behav..

[35] Alarcón G., Cservenka A., Rudolph M.D., Fair D.A., Nagel B.J. (2015). Developmental Sex Differences in Resting State Functional Connectivity of Amygdala Sub-Regions. Neuroimage.

[36] Shansky R.M. (2020). Sex Differences in Amygdala Structure and Function: From Rodents to Humans. Handbook of Behavioral Neuroscience.

[37] Lauretta R., Sansone M., Sansone A., Romanelli F., Appetecchia M. (2018). Gender in Endocrine Diseases: Role of Sex Gonadal Hormones. Int. J. Endocrinol..

[38] Peper J.S., van den Heuvel M.P., Mandl R.C.W., Hulshoff Pol H.E., van Honk J. (2011). Sex Steroids and Connectivity in the Human Brain: A Review of Neuroimaging Studies. Psychoneuroendocrinology.

[39] Bos P.A., Hermans E.J., Ramsey N.F., van Honk J. (2012). The Neural Mechanisms by Which Testosterone Acts on Interpersonal Trust. Neuroimage.

[40] Votinov M., Wagels L., Hoffstaedter F., Kellermann T., Goerlich K.S., Eickhoff S.B., Habel U. (2020). Effects of Exogenous Testosterone Application on Network Connectivity within Emotion Regulation Systems. Sci. Rep..

[41] Van Wingen G., Mattern C., Verkes R.J., Buitelaar J., Fernández G. (2010). Testosterone Reduces Amygdala-Orbitofrontal Cortex Coupling. Psychoneuroendocrinology.

[42] Heany S.J., Bethlehem R.A.I., van Honk J., Bos P.A., Stein D.J., Terburg D. (2018). Effects of Testosterone Administration on Threat and Escape Anticipation in the Orbitofrontal Cortex. Psychoneuroendocrinology.

[43] Peters S., Jolles D.J., Duijvenvoorde A.C.K.V., Crone E.A., Peper J.S. (2015). The Link between Testosterone and Amygdala-Orbitofrontal Cortex Connectivity in Adolescent Alcohol Use. Psychoneuroendocrinology.

[44] Volman I., Toni I., Verhagen L., Roelofs K. (2011). Endogenous Testosterone Modulates Prefrontal-Amygdala Connectivity during Social Emotional Behavior. Cereb. Cortex.

[45] Cahill L. (2006). Why Sex Matters for Neuroscience. Nat. Rev. Neurosci..

[46] McCarthy M.M., Arnold A.P., Ball G.F., Blaustein J.D., De Vries G.J. (2012). Sex Differences in the Brain: The Not so Inconvenient Truth. J. Neurosci..

[47] Mauvais-Jarvis F., Bairey Merz N., Barnes P.J., Brinton R.D., Carrero J.J., DeMeo D.L., De Vries G.J., Epperson C.N., Govindan R., Klein S.L. (2020). Sex and Gender: Modifiers of Health, Disease, and Medicine. Lancet.

[48] Cowell P.E., Kostianovsky D.J., Gur R.C., Turetsky B.I., Gur R.E. (1996). Sex Differences in Neuroanatomical and Clinical Correlations in Schizophrenia. Am. J. Psychiatry.

[49] Power J.D., Barnes K.A., Snyder A.Z., Schlaggar B.L., Petersen S.E. (2012). Spurious but Systematic Correlations in Functional Connectivity MRI Networks Arise from Subject Motion. Neuroimage.

[50] Satterthwaite T.D., Elliott M., Gerraty R., Ruparel K., Loughead J., Calkin M.E., Eickhoff S.B., Hakonarson H., Gur R.C., Gur R.E. (2013). An Improved Framework for Confound Regression and Filtering for Control of Motion Artifact in the Preprocessing of Resting-State Functional Connectivity Data. Neuroimage.

[51] Watson D., Clark L.A., Tellegen A. (1988). Development and Validation of Brief Measures of Positive and Negative Affect: The PANAS Scales. J. Pers. Soc. Psychol..

[52] Radke S., Seidel E.M., Boubela R.N., Thaler H., Metzler H., Kryspin-Exner I., Moser E., Habel U., Derntl B. (2018). Immediate and Delayed Neuroendocrine Responses to Social Exclusion in Males and Females. Psychoneuroendocrinology.

[53] Kogler L., Seidel E.-M., Metzler H., Thaler H., Boubela R.N., Pruessner J.C., Kryspin-Exner I., Gur R.C., Windischberger C., Moser E. (2017). Impact of Self-Esteem and Sex on Stress Reactions. Sci. Rep..

[54] Bürger Z., Müller V.I., Hoffstaedter F., Habel U., Gur R.C., Windischberger C., Moser E., Derntl B., Kogler L. (2023). Stressor-Specific Sex Differences in Amygdala-Frontal Cortex Networks. J. Clin. Med..

[55] McCrae R.R., Costa P.T. (2004). A Contemplated Revision of the NEO Five-Factor Inventory. Pers. Individ. Dif..

[56] Bem S.L. (1974). The Measurement of Psychological Androgyny. J. Consult. Clin. Psychol..

[57] Arregger A.L., Contreras L.N., Tumilasci O.R., Aquilano D.R., Cardoso E.M.L. (2007). Salivary Testosterone: A Reliable Approach to the Diagnosis of Male Hypogonadism. Clin. Endocrinol..

[58] Vittek J., Hommedieu D., Gordon G.G., Rappaport S.C., Southren A.L. (1985). Direct Radioimmunoassay (RIA) of Salivary Testosterone: Correlation with Free and Total Serum Testosterone. Life Sci..

[59] Holmes C.J., Hoge R., Collins L., Woods R., Toga A.W. (1998). Enhancement of MR Images Using Registration for Signal Averaging. J. Comput. Assist. Tomogr..

[60] Ashburner J., Friston K.J. (2005). Unified Segmentation. Neuroimage.

[61] Weissenbacher A., Kasess C., Gerstl F., Lanzenberger R., Moser E., Windischberger C. (2009). Correlations and Anticorrelations in Resting-State Functional Connectivity MRI: A Quantitative Comparison of Preprocessing Strategies. Neuroimage.

[62] Eickhoff S.B., Stephan K.E., Mohlberg H., Grefkes C., Fink G.R., Amunts K., Zilles K. (2005). A New SPM Toolbox for Combining Probabilistic Cytoarchitectonic Maps and Functional Imaging Data. Neuroimage.

[63] Laird A.R., Lancaster J.L., Fox P.T. (2005). BrainMap: The Social Evolution of a Human Brain Mapping Database. Neuroinformatics.

[64] Turner J., Laird A. (2012). The Cognitive Paradigm Ontology: Design and Application. Neuroinformatics.

[65] Müller V.I., Cieslik E.C., Laird A.R., Fox P.T., Eickhoff S.B. (2013). Dysregulated Left Inferior Parietal Activity in Schizophrenia and Depression: Functional Connectivity and Characterization. Front. Hum. Neurosci..

[66] Kogler L., Müller V.I., Chang A., Eickhoff S.B., Fox P.T., Gur R.C., Derntl B. (2015). Psychosocial versus Physiological Stress—Meta-Analyses on the Deactivations and Activations of the Neural Correlates of Stress Reactions. Neuroimage.

[67] Kirschbaum C., Wüst S., Hellhammer D. (1992). Consistent Sex Differences in Cortisol Responses to Psychological Stress. Psychosom. Med..

[68] Vermeersch H., T’Sjoen G., Kaufman J.M., Vincke J., Van Houtte M. (2010). Gender Ideology, Same-Sex Peer Group Affiliation and the Relationship between Testosterone and Dominance in Adolescent Boys and Girls. J. Biosoc. Sci..

[69] Seidel E., Silani G., Metzler H., Thaler H., Lamm C., Gur R., Kryspin-Exner I., Habel U., Derntl B. (2013). The Impact of Social Exclusion vs. Inclusion on Subjective and Hormonal Reactions in Females and Males. Psychoneuroendocrinology.

[70] Fernández-Guasti A., Kruijver F.P.M., Fodor M., Swaab D.F. (2000). Sex Differences in the Distribution of Androgen Receptors in the Human Hypothalamus. J. Comp. Neurol..

[71] Celec P., Ostatníková D., Hodosy J. (2015). On the Effects of Testosterone on Brain Behavioral Functions. Front. Neurosci..

[72] Höfer P., Lanzenberger R., Kasper S. (2013). Testosterone in the Brain: Neuroimaging Findings and the Potential Role for Neuropsychopharmacology. Eur. Neuropsychopharmacol..

[73] Güntürkün O., Ströckens F., Ocklenburg S. (2020). Brain Lateralization: A Comparative Perspective. Physiol. Rev..

[74] Pletzer B., Harris T. (2018). Sex Hormones Modulate the Relationship between Global Advantage, Lateralization, and Interhemispheric Connectivity in a Navon Paradigm. Brain Connect..

[75] Toga A.W., Thompson P.M. (2003). Mapping Brain Asymmetry. Nat. Rev. Neurosci..

[76] Fang H., Li X., Wu Y., Peng W. (2020). Single Dose Testosterone Administration Modulates the Temporal Dynamics of Distractor Processing. Psychoneuroendocrinology.

[77] Pintzka C.W.S., Evensmoen H.R., Lehn H., Håberg A.K. (2016). Changes in Spatial Cognition and Brain Activity after a Single Dose of Testosterone in Healthy Women. Behav. Brain Res..

[78] Janowsky J.S. (2006). Thinking with Your Gonads: Testosterone and Cognition. Trends Cogn. Sci..

[79] Schöning S., Engelien A., Kugel H., Schäfer S., Schiffbauer H., Zwitserlood P., Pletziger E., Beizai P., Kersting A., Ohrmann P. (2007). Functional Anatomy of Visuo-Spatial Working Memory during Mental Rotation Is Influenced by Sex, Menstrual Cycle, and Sex Steroid Hormones. Neuropsychologia.

[80] Pletzer B., Petasis O., Cahill L. (2014). Switching between Forest and Trees: Opposite Relationship of Progesterone and Testosterone to Global-Local Processing. Horm. Behav..

[81] Schutter D.J.L.G., Meuwese R., Bos M.G.N., Crone E.A., Peper J.S. (2017). Exploring the Role of Testosterone in the Cerebellum Link to Neuroticism: From Adolescence to Early Adulthood. Psychoneuroendocrinology.

[82] Courvoisier D.S., Renaud O., Geiser C., Paschke K., Gaudy K., Jordan K. (2013). Sex Hormones and Mental Rotation: An Intensive Longitudinal Investigation. Horm. Behav..

[83] Genon S., Li H., Fan L., Müller V.I., Cieslik E.C., Hoffstaedter F., Reid A.T., Langner R., Grefkes C., Fox P.T. (2017). The Right Dorsal Premotor Mosaic: Organization, Functions, and Connectivity. Cereb. Cortex.

[84] Choi J.C., Yi D.J., Han B.S., Lee P.H., Kim J.H., Kim B.H. (2011). Placebo Effects on Analgesia Related to Testosterone and Premotor Activation. Neuroreport.

[85] Glinka K., Staudinger U.M., Voelcker-Rehage C., Godde B. (2020). Neural Processing of Arousing Emotional Information Is Associated with Executive Functioning in Older Adults. Emotion.

[86] Faul L., Knight L.K., Espay A.J., Depue B.E., LaFaver K. (2020). Neural Activity in Functional Movement Disorder after Inpatient Rehabilitation. Psychiatry Res. Neuroimaging.

[87] Bos P.A., Panksepp J., Bluthe R.M., van Honk J. (2012). Acute Effects of Steroid Hormones and Neuropeptides on Human Social-Emotional Behavior: A Review of Single Administration Studies. Front. Neuroendocrinol..

[88] Ulrich M., Niemann J., Boland M., Kammer T., Niemann F., Grön G. (2018). The Neural Correlates of Flow Experience Explored with Transcranial Direct Current Stimulation. Exp. Brain Res..

[89] Dedoncker J., Brunoni A.R., Baeken C., Vanderhasselt M.A. (2016). A Systematic Review and Meta-Analysis of the Effects of Transcranial Direct Current Stimulation (tDCS) Over the Dorsolateral Prefrontal Cortex in Healthy and Neuropsychiatric Samples: Influence of Stimulation Parameters. Brain Stimulat..

[90] Veldema J. (2023). Non-Invasive Brain Stimulation and Sex/Polypeptide Hormones in Reciprocal Interactions: A Systematic Review. Biomedicines.

[91] Nguyen T.V., McCracken J.T., Albaugh M.D., Botteron K.N., Hudziak J.J., Ducharme S. (2016). A testosterone-related structural brain phenotype predicts aggressive behavior from childhood to adulthood. Psychoneuroendocrinology.

[92] Zuloaga D.G., Puts D.A., Jordan C.L., Breedlove S.M. (2008). The role of androgen receptors in the masculinization of brain and behavior: What we’ve learned from the testicular feminization mutation. Horm. Behav..

[93] Pletzer B. (2014). Sex-Specific Strategy Use and Global-Local Processing: A Perspective toward Integrating Sex Differences in Cognition. Front. Neurosci..

[94] Jordan K., Wüstenberg T., Heinze H.-J., Peters M., Jäncke L. (2002). Women and Men Exhibit Different Cortical Activation Patterns during Mental Rotation Tasks. Neuropsychologia.

[95] Butler T., Imperato-McGinley J., Pan H., Voyer D., Cordero J., Zhu Y.S., Stern E., Silbersweig D. (2006). Sex Differences in Mental Rotation: Top-down versus Bottom-up Processing. Neuroimage.

[96] Hausmann M., Schoofs D., Rosenthal H.E.S., Jordan K. (2009). Interactive Effects of Sex Hormones and Gender Stereotypes on Cognitive Sex Differences—A Psychobiosocial Approach. Psychoneuroendocrinology.

[97] Zell E., Krizan Z., Teeter S.R. (2015). Evaluating Gender Similarities and Differences Using Metasynthesis. Am. Psychol..

[98] Finkelstein M., Weidenfeld J., Ne’eman Y., Samuni A., Mizrachi Y., Ben-Uzilio R. (1981). Comparative Studies of the Aromatization of Testosterone and Epitestosterone by Human Placental Aromatase. Endocrinology.

[99] Wright F., Giacomini M., Riahi M., Mowszowicz I., Bardin C.W., Milgröm E., Mauvais-Jarvis P. (1983). Antihormone Activity of Progesterone and Progestins. Progesterone and Progestins.

[100] Bozdag G., Mumusoglu S., Zengin D., Karabulut E., Yildiz B.O. (2016). The prevalence and phenotypic features of polycystic ovary syndrome: A systematic review and meta-Analysis. Hum. Reprod..

[101] Sebastián-Tirado A., Félix-Esbrí S., Forn C., Sanchis-Segura C. (2023). Are Gender-Science Stereotypes Barriers for Women in Science, Technology, Engineering, and Mathematics? Exploring When, How, and to Whom in an Experimentally-Controlled Setting. Front. Psychol..

[102] Stoevenbelt A.H., Wicherts J.M., Flore P.C., Phillips L.A.T., Pietschnig J., Verschuere B., Voracek M., Schwabe I. (2022). Are Speeded Tests Unfair? Modeling the Impact of Time Limits on the Gender Gap in Mathematics. Educ. Psychol. Meas..

[103] Engman J., Sundström-Poromaa I., Moby L., Wikström J., Fredrikson M., Gingnell M. (2018). Hormonal Cycle and Contraceptive Effects on Amygdala and Salience Resting-State Networks in Women with Previous Affective Side Effects on the Pill. Neuropsychopharmacology.

[104] Engman J., Linnman C., Van Dijk K.R., Milad M.R. (2016). Amygdala subnuclei resting-state functional connectivity sex and estrogen differences. Psychoneuroendocrinology.

[105] Hidalgo-Lopez E., Noachtar I., Pletzer B. (2023). Hormonal contraceptive exposure relates to changes in resting state functional connectivity of anterior cingulate cortex and amygdala. Front. Endocrinol..

[106] Cooper M.A., Clinard C.T., Dulka B.N., Grizzell J.A., Loewen A.L., Campbell A.V., Adler S.G. (2021). Gonadal Steroid Hormone Receptors in the Medial Amygdala Contribute to Experience-Dependent Changes in Stress Vulnerability. Psychoneuroendocrinology.

[107] Josephs R.A., Telch M.J., Hixon J.G., Evans J.J., Lee H., Knopik V.S., McGeary J.E., Hariri A.R., Beevers C.G. (2012). Genetic and hormonal sensitivity to threat: Testing a serotonin transporter genotype × testosterone interaction. Psychoneuroendocrinology.

[108] Maseroli E., Vignozzi L. (2022). Are Endogenous Androgens Linked to Female Sexual Function? A Systemic Review and Meta-Analysis. J. Sex. Med..

