# Supplementary material for: Testosterone and the Amygdala’s Functional Connectivity in Women and Men
Source: J Clin Med. 2023 Oct 13;12(20):6501. doi: 10.3390/jcm12206501 (PMC10607739; doi:10.3390/jcm12206501)
Supplement: Supplementary file 1 [file jcm-12-06501-s001.zip › jcm-2513145-supplementary.pdf]

Table S1. Multiple linear regressions (standardized beta coefficients) for sex, resting-state functional connectivity (rsFC) of the right amygdala with the right MOG and the right SFG, and their interactions (IA), as predictors of social behavior.

	Sex	rsFC [right Amy – right MOG]	rsFC [right Amy – right SFG]	IA rsFC [right Amy – right MOG] – sex	IA rsFC [right Amy – right SFG] - sex	R^2	*p*
Neuroticism	0.249*	0.245	-0.233	-0.98	0.105	0.111	n.s.
Extraversion	0.107	-0.126	0.144	0.091	-0.215	0.037	n.s.
Openness	0.305*	-0.001	0.109	0.203	-0.153	0.150	0.029*
Agreeableness	0.381*	-0.053	0.136	0.039	0.008	0.171	0.014*
Conscientiousness	0.136	-0.038	0.048	-0.014	0.078	0.035	n.s.
Masculinity	-0.029	-0.141	0.098	0.221	-0.165	0.042	n.s.
Femininity	0.207	0.084	-0.058	-0.012	-0.040	0.054	n.s.
Androgyny	0.163	0.266	-0.135	-0.292*	0.098	0.087	n.s.

Note: Determination coefficients (R^2) and significance (*p*) indicate the goodness-of-fit for the regression model. *p<0.05. n.s.=not significant.

Table S2. Multiple linear regressions (standardized beta coefficients) for testosterone (testo), resting-state functional connectivity (rsFC) of the right amygdala with the right MOG and the right SFG, and their interactions (IA), as predictors of social behavior (NEO-FFI, BSRI).

	Testosterone	rsFC [right Amy – right MOG]	rsFC [right Amy – right SFG]	IA rsFC [right Amy – right MOG] – testo	IA rsFC [right Amy – right SFG] - testo	R^2	*p*
Neuroticism	0.006	0.186	-0.114	-0.042	-0.054	0.047	n.s.
Extraversion	-0.193	0.011	-0.061	0.011	0.040	0.036	n.s.
Openness	-0.256*	0.220	-0.066	0.009	0.087	0.084	n.s.
Agreeableness	-0.385*	0.083	0.095	0.077	-0.006	0.169	0.015*
Conscientiousness	-0.202	0.003	0.086	0.012	-0.011	0.053	n.s.
Masculinity	-0.177	0.069	-0.101	-0.1386	0.167	0.058	n.s.
Femininity	3.613	5.998	5.420	19.323	15.153	0.030	n.s.
Androgyny	0.085	0.051	0.008	0.144	-0.129	0.039	n.s.

Note: Determination coefficients (R^2) and significance (*p*) indicate the goodness-of-fit for the regression model. *p<0.05. n.s.=not significant.
